# Increasing the Number of Thyroid Lesions Classes in Microarray Analysis Improves the Relevance of Diagnostic Markers

**DOI:** 10.1371/journal.pone.0007632

**Published:** 2009-10-29

**Authors:** Jean-Fred Fontaine, Delphine Mirebeau-Prunier, Mahatsangy Raharijaona, Brigitte Franc, Stephane Triau, Patrice Rodien, Olivier Goëau-Brissonniére, Lucie Karayan-Tapon, Marielle Mello, Rémi Houlgatte, Yves Malthiery, Frédérique Savagner

**Affiliations:** 1 Max Delbrück Center for Molecular Medicine, Berlin, Germany; 2 INSERM, UMR 694, Angers, France; 3 Université d'Angers, Angers, France; 4 CHU Angers, Laboratoire de Biochimie, Angers, France; 5 INSERM, UMR 915, l'institut du Thorax, Nantes, France; 6 Université de Nantes, Nantes, France; 7 Hôpital A Paré, Laboratoire d'Anatomie Pathologique, Boulogne, France; 8 CHU Angers, Laboratoire de Pathologie Cellulaire et Tissulaire, Angers, France; 9 CHU Angers, Département Endocrinologie-Diabétologie-Nutrition, Angers, France; 10 Hôpital A Paré, Service de Chirurgie vasculaire, Boulogne, France; 11 Université de Poitiers, EA 3805, Poitiers, France; 12 INSERM, UMR136, Marseille, France; Texas Tech University Health Sciences Center, United States of America

## Abstract

**Background:**

Genetic markers for thyroid cancers identified by microarray analysis have offered limited predictive accuracy so far because of the few classes of thyroid lesions usually taken into account. To improve diagnostic relevance, we have simultaneously analyzed microarray data from six public datasets covering a total of 347 thyroid tissue samples representing 12 histological classes of follicular lesions and normal thyroid tissue. Our own dataset, containing about half the thyroid tissue samples, included all categories of thyroid lesions.

**Methodology/Principal Findings:**

Classifier predictions were strongly affected by similarities between classes and by the number of classes in the training sets. In each dataset, sample prediction was improved by separating the samples into three groups according to class similarities. The cross-validation of differential genes revealed four clusters with functional enrichments. The analysis of six of these genes (APOD, APOE, CLGN, CRABP1, SDHA and TIMP1) in 49 new samples showed consistent gene and protein profiles with the class similarities observed. Focusing on four subclasses of follicular tumor, we explored the diagnostic potential of 12 selected markers (CASP10, CDH16, CLGN, CRABP1, HMGB2, ALPL2, ADAMTS2, CABIN1, ALDH1A3, USP13, NR2F2, KRTHB5) by real-time quantitative RT-PCR on 32 other new samples. The gene expression profiles of follicular tumors were examined with reference to the mutational status of the Pax8-PPARγ, TSHR, GNAS and NRAS genes.

**Conclusion/Significance:**

We show that diagnostic tools defined on the basis of microarray data are more relevant when a large number of samples and tissue classes are used. Taking into account the relationships between the thyroid tumor pathologies, together with the main biological functions and pathways involved, improved the diagnostic accuracy of the samples. Our approach was particularly relevant for the classification of microfollicular adenomas.

## Introduction

Over the past few years, the use of microarray technologies has contributed to the identification of new markers for the diagnosis and prognosis of human tumors. Cancer research usually involves the study of a single class of tumor and the corresponding normal tissue. Increasing the number of binary studies does not necessarily improve the relevance of the molecular signature. Thus, a meta-analysis comparing 40 types of cancer in various tissues relative to their normal counterpart allowed the identification of a common signature essential to carcinogenesis but may fail to distinguish between different classes of tumor affecting a given organ, which may therefore have specific prognoses [1]. Moreover, the same signature may also appear in a variety of other cellular contexts such as inflammatory processes.

Thyroid nodules are extremely common in the adult population, but less than 20% of the nodules are malignant [2]. Papillary thyroid carcinoma (PTC), diagnosed on the basis of characteristic nuclear features, is the most frequent malignant thyroid tumor. According to the 2004 WHO report, the diagnosis of minimal invasive follicular thyroid carcinoma (FTC) is problematic because of its morphological and molecular similarities to benign follicular thyroid adenoma (FTA) [3]. Moreover, atypical or oncocytic features render the differential diagnosis of follicular tumors difficult on histologic examination and call for new molecular or biological markers [4], [5]. To date, microarray analyses of thyroid tumors have essentially compared two classes of tissue [6]–[11]. These studies have either searched for specific markers by comparing a particular class of thyroid tumor to the corresponding normal tissue, or looked for markers of malignancy by examining the most frequent benign and malignant classes of thyroid tumor (usually the FTA and PTC classes). The predictive accuracy of the markers identified is therefore rather limited with regard to tumors belonging to other classes. For example, the CITED1 gene, which was claimed to be a significant marker distinguishing PTC from normal tissue [9] turned out to be less specific when data from FTC samples were included [8]. Furthermore, this gene did not even figure among the 42 best PTC marker genes detected by a meta-analysis that included benign tumors [12]. Meta-analyses may increase not only the number of classes required to define more relevant markers but also increase the imbalance in the representation of some classes. In a recent analysis, cross-validated marker genes from 21 studies differentiated benign from malignant thyroid tissues [13]. However, the majority of these were pertinent to the diagnosis of PTC since these thyroid cancers accounted for more than 40% of the samples studied. This highlights the bias due to the recruitment of thyroid samples for microarray studies and the consequent failure in identifying classifiers for clinical applications despite the large number of analyses exploited.

Few studies have simultaneously compared more than four types of thyroid tissue [5], [14]–[17]. In one of these [15], we were able to refine the diagnosis of tumors of uncertain malignancy by the simultaneous analysis of eight types of thyroid tissue. These promising results encouraged further research on relevant markers of thyroid tumors, taking into account more classes and subclasses of interest, as some authors have recommended [18]. In the present study, we have explored gene-expression signatures from the majority of the differentiated follicular pathologies of thyroid tissue. We have used published data or data available from the Gene Expression Omnibus (GEO) of the U. S. National Center for Biotechnology Information, as well as specifically generated data, to simultaneously analyze several datasets containing information on various pathologies, including datasets with many histological subclasses. Our analysis, covering the major subtypes of thyroid tissue, may be expected to enhance microarray data concerning thyroid tumors and lead to the definition of more reliable tissue-specific markers for thyroid lesions.

## Results

We have simultaneously analyzed 347 thyroid tissue samples from six datasets (Table 1). The Fontaine dataset, generated by our own laboratory [15], comprised 166 thyroid tissue samples, i.e. about half the total number of samples.

**Table 1:** Datasets, classes and samples.

*Datasets*		*Fontaine et al. 2008*	*Giordano et al.2006*	*Weber et al.2005*	*Jarzab et al. 2005*	*He et al. 2005*	*Reyes et al. 2006*
*Microarray platform*		Single channel cDNA microarrays	Affymetrix GeneChip HG U133A	Affymetrix GeneChip HG U133A	Affymetrix GeneChip HG U133A	Affymetrix GeneChip HG U133 Plus 2.0	Affymetrix GeneChip HG U133 Plus 2.0
*(Probes)*		*(9,216)*	*(22,283)*	*(22,283)*	*(22,283)*	*(54,681)*	*(54,681)*
*Tissue samples*	*Symbol*	166	93	24	32	18	14
Wild Type tissue	WT	24	4	-	16	9	7
Autoimmune Thyroiditis	AT	5	-	-	-	-	-
Grave's Disease	GD	5	-	-	-	-	-
Follicular Thyroid Adenoma	FTA	26	10	12	-	-	-
Microfollicular Thyroid Adenoma	FTAb	17	-	-	-	-	-
Multi Nodular Goitre	MNG	24	-	-	-	-	-
Follicular Thyroid Carcinoma	FTC	3	-	6	-	-	-
* FTC with PAX8/PPARγ fusion*	FTC+	-	7	-	-	-	-
* FTC without PAX8/PPARγ fusion*	FTC-	-	6	-	-	-	-
Oncocytic Thyroid Adenoma	OTA	30	7	-	-	-	-
Oncocytic Thyroid Carcinoma	OTC	4	8	6	-	-	-
Oncocytic Tumour of Uncertain	OTUM	5	-	-	-	-	-
Malignancy							
Papillary Thyroid Carcinoma	PTC	13	51	-	16	9	7
Tumour of Uncertain Malignancy	TUM	10	-	-	-	-	-

### Molecular classification of thyroid pathologies

**Figure 1:**
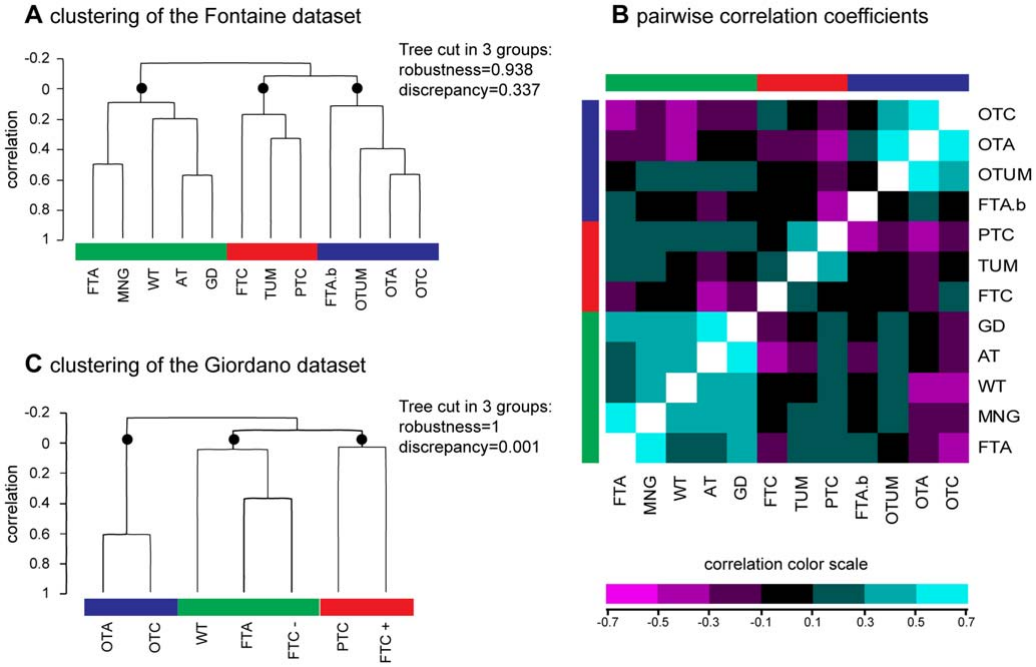
Molecular classification of thyroid tissues. The centroid signatures of the thyroid tissues were compared to each other in the Fontaine dataset generated by our laboratory [15], and compared to the Giordano dataset [5]. Centroids were defined as the mean gene-expression signature of each tissue class over all the genes. Panel A shows the hierarchical clustering of the tissues in the Fontaine dataset. The dendrogram can be cut into three robust branches (black circles) defining three groups of classes. The groups contain benign lesions and normal tissue (green boxes), malignant tumors (red boxes), or oncocytic tumors and microfollicular adenomas (blue boxes). Panel B shows pairwise correlation coefficients between the tissue classes from the Fontaine dataset. The heatmap represents a symmetrical matrix of color-coded correlation coefficients. Tissue classes are ordered as in the hierarchical clustering and marked with the same three colors (green, red and blue). Panel C shows the hierarchical clustering of the tissues in the Giordano dataset. The dendrogram can be cut into three robust branches (black circles) defining three groups of classes. The groups contain heterogeneous lesions and normal tissue (green boxes), malignant tumors (red boxes), or oncocytic tumors (blue boxes). The two datasets show three groups containing similar tissue classes. WT: wild type tissue; PTC: papillary thyroid carcinoma; FTA: macrofollicular thyroid adenoma; FTAb: microfollicular thyroid adenoma; GMN, multinodular goiter; FTC: follicular thyroid carcinoma (FTC+: with Pax8/PPARγ translocation; FTC-: without Pax8/PPARγ translocation); OTA: oncocytic thyroid adenoma; OTC: oncocytic thyroid carcinoma; TUM: tumor of uncertain malignancy; OTUM: oncocytic tumor of uncertain malignancy; GD: Grave's disease; and AT: autoimmune thyroïditis.

To analyze the global classification of the follicular thyroid lesions in the Fontaine dataset, we defined the centroid of each class of tissue by a composite signature of mean gene-expression levels. The hierarchical clustering of the class centroids (Figure 1A) revealed three groups. The first group comprised benign thyroid lesions and normal tissues (green boxes for FTA, MNG, WT, AT, and GD); the second contained malignant lesions (red boxes for FTC, TUM and PTC); and the third included oncocytic tumors and microfollicular adenomas (blue boxes for FTAb, OTUM, OTA and OTC). These groups, supported by a high robustness index of 0.938 and a discrepancy index of 0.337, are respectively referred to as the ‘benign’, ‘malignant’ and ‘oncocytic’ groups. The three groups, cross-validated in the Giordano dataset (Figure 1C), presented almost perfect robustness ( = 1) and discrepancy indices ( = 0.001). This dataset showed a highly correlated oncocytic group (OTA, OTC, correlation >0.6) and a group of malignant tumors (PTC, FTC+). The third group, predominantly composed of benign tumors and normal tissues (WT, FTA), also included the FTC- group.

We computed the prediction accuracy for the benign, malignant and oncocytic groups of thyroid tissue classes as defined above. The signature of each group was trained *versus* all the other samples, and its accuracy was compared with the predictive accuracy of individual lesions (Table 2). In both datasets, the positive accuracy for each group was greater than the best accuracy reported for an individual lesion within the group. The high proportion of PTC (79%) in the malignant group from the Giordano dataset may explain the good accuracy for this group despite the rate of discrepancy shown in the Figure 1C. The separation of the samples into three groups on the basis of class similarities led to improved sample prediction in the two datasets.

**Table 2:** Prediction of samples by considering three groups of tissue classes. Values can be compared to the range of accuracies for individual lesions that composed each group.

	Fontaine dataset		Giordano dataset	
	Group	Individual lesions	Group	Individual lesions
Benign group	0,79	0.11–0.71	0,76	0.31–0.47
Malignant group	0,69	0.24–0.67	0,93	0.93–0.94
Oncocytic group	0,75	0.02–0.64	0,86	0.52–0.62

The matrix of 66 pairwise correlation coefficients between the classes (Figure 1B) allowed a detailed observation of the similarities and dissimilarities in the Fontaine dataset. All the classes in the benign/normal group were positively correlated with each other. Some pairs of classes were highly correlated (correlation coefficient >0.4), e.g. FTA with MNG, and AT with GD. All the classes in the malignant group were positively correlated with each other. The TUM class had a stronger correlation with PTC than with FTC (respectively 0.354 and 0.274). This similarity between TUM and PTC has previously been demonstrated within this dataset and with independent samples [15]. In the oncocytic group, the classes of oncocytic tumors were positively correlated with each other, except for the FTAb class. Finally, we were able to identify 220 non-redundant genes for the molecular classification of each of the 11 subcategories of follicular thyroid pathologies explored (Table 3).

**Table 3:** Classifier genes from the Fontaine dataset (n = 220 genes).

Symbol	Clone ID	Class	Cross validation support (%)	Parametric p-value	t-value	Fold-change
ABCB9	36371	AT	100	1,00E-07	6,81	3,28
SEP-02	729542	AT	100	2,67E-01	−1,11	0,75
SEP-05	448163	AT	100	1,00E-07	7,03	4,96
AAAS	740617	AT	99	3,00E-07	5,36	3,34
ABCA3	52740	AT	97	4,99E-01	0,68	1,13
ABCA8	284828	AT	97	1,86E-01	−1,33	0,74
ABCB1	1837488	AT	97	2,26E-01	−1,22	0,72
ABCB8	238705	AT	97	3,51E-01	−0,94	0,84
ABCC3	208097	AT	96	1,54E-01	1,43	1,64
ABCF2	1086914	AT	96	1,00E-07	6,91	3,37
BATF	1929371	AT	83	1,00E-07	10,52	6,03
C18orf51	742649	AT	62	1,00E-07	9,87	7,47
CARD11	665651	AT	46	1,00E-07	10,85	4,72
CBFA2T2	43629	AT	39	1,00E-07	10,18	10,03
CD38	1352408	AT	29	1,00E-07	10,18	7,22
CD79A	1056782	AT	25	1,00E-07	11,14	8,58
CD79B	155717	AT	25	1,00E-07	11,91	10,51
CXCL6	2108870	AT	14	1,00E-07	10,13	4,29
EPPB9	668454	AT	7	1,00E-07	9,67	9,03
DKFZp667B0210	1844689	AT	3	1,00E-07	9,30	4,83
PLXNB2	1420676	FTA	65	6,00E-07	5,21	1,70
TBC1D17	739955	FTA	62	4,00E-07	5,27	1,56
ZNF76	745003	FTA	59	8,00E-07	5,15	1,51
KIAA1196	738938	FTA	52	1,40E-06	5,01	1,52
ASGR1	25883	FTA	48	1,25E-05	4,51	1,75
BCL2L11	300194	FTA	42	6,00E-06	4,68	1,42
DAP	725371	FTA	41	6,20E-06	4,67	1,51
CABIN1	1844968	FTA	40	1,12E-05	4,53	1,59
FLJ14981	24532	FTA	31	1,84E-04	3,83	1,50
RAD51L1	295412	FTA	30	4,23E-05	4,21	1,66
TSPAN1	376003	FTA	26	3,52E-05	4,25	1,57
IMAGE:745465	745465	FTA	25	2,58E-02	2,25	1,24
PLCG1	1174287	FTA	25	8,36E-05	−4,04	0,67
COL18A1	359202	FTA	24	3,20E-05	4,28	1,60
RPS6KA2	22711	FTA	23	3,77E-05	4,24	1,57
CaMKIINalpha	173820	FTA	22	9,52E-04	3,37	1,35
KLF1	208991	FTA	16	3,01E-03	3,01	1,49
BAT3	24392	FTA	13	8,11E-04	3,41	1,42
PDK4	487379	FTA	11	1,00E-02	−2,61	0,70
IMAGE:24065	24065	FTA	6	4,52E-04	−3,58	0,75
KRTHB5	364569	FTAb	86	1,00E-07	6,02	1,94
METRN	487475	FTAb	86	1,00E-07	5,68	2,15
DCI	667892	FTAb	74	6,00E-07	5,19	1,63
IFI30	740931	FTAb	68	4,90E-06	−4,73	0,41
ALCAM	172828	FTAb	64	1,21E-05	−4,51	0,68
ADAMTS2	364844	FTAb	55	1,61E-04	3,86	1,41
ETF1	146976	FTAb	54	1,12E-04	3,96	1,54
TP53I11	667514	FTAb	44	1,22E-04	3,94	1,94
IMAGE:487086	487086	FTAb	42	1,52E-04	−3,88	0,67
IMAGE:666315	666315	FTAb	35	5,63E-04	3,52	1,52
GRB10	564994	FTAb	33	1,80E-04	3,83	1,42
ISG20	740604	FTAb	33	3,51E-05	−4,26	0,60
SPARC	267358	FTAb	33	1,09E-04	3,97	1,47
HAS3	667533	FTAb	28	1,07E-04	3,97	1,52
USP13	666007	FTAb	20	9,96E-05	3,99	1,56
KRT4	1173570	FTAb	13	2,87E-04	3,71	1,65
LHFP	591534	FTAb	9	2,24E-03	−3,11	0,74
PCDHB15	730593	FTAb	9	1,06E-03	−3,33	0,74
TPM2	740620	FTAb	9	9,35E-03	2,63	1,45
DKFZp434M202	743118	FTAb	2	5,77E-03	−2,80	0,76
C22orf9	667250	FTC	89	2,00E-07	−5,43	0,34
SUCLG2	687551	FTC	83	7,00E-07	−5,18	0,27
LAPTM4A	726684	FTC	77	4,00E-07	−5,26	0,24
KIAA1576	743426	FTC	76	1,00E-07	5,70	3,33
NR2F2	72744	FTC	65	2,60E-06	4,87	3,11
CHST3	665327	FTC	62	2,90E-06	−4,85	0,32
PAI-RBP1	236055	FTC	55	5,30E-06	−4,71	0,29
IMAGE:729896	729896	FTC	54	3,00E-07	5,34	3,22
ZBTB16	2467442	FTC	52	4,72E-05	4,18	4,68
ZNF364	471834	FTC	52	5,80E-06	−4,69	0,39
GHITM	25077	FTC	50	5,30E-06	−4,71	0,31
LRP8	415554	FTC	49	1,43E-04	3,90	2,74
CEECAM1	666784	FTC	45	2,67E-04	3,73	2,22
HIST1H4C	682451	FTC	43	1,57E-05	−4,45	0,25
TIE1	743043	FTC	35	5,46E-05	4,14	2,52
SH3RF2	744797	FTC	22	5,37E-03	2,82	2,18
MGC5395	238840	FTC	15	5,07E-04	3,55	2,66
EMX2	365121	FTC	14	6,95E-03	−2,73	0,51
LRRC28	26519	FTC	13	1,51E-03	−3,23	0,40
GFRA3	2716748	FTC	11	4,52E-03	2,88	1,93
TTC19	136366	GD	72	1,57E-04	3,87	1,87
ATP6V1G2	726424	GD	61	1,91E-04	3,82	2,22
IMAGE:23817	23817	GD	61	3,65E-04	−3,64	0,52
IMAGE:178161	178161	GD	52	6,99E-04	3,46	2,53
BDKRB2	665674	GD	50	6,16E-03	2,78	2,15
IMAGE:744385	744385	GD	48	1,37E-02	2,49	1,70
LOC150837	258698	GD	47	5,84E-04	3,51	1,81
SSX1	262894	GD	47	5,97E-04	3,50	1,76
DHX57	24623	GD	46	1,69E-03	−3,19	0,59
RAI14	731711	GD	46	8,19E-03	2,68	1,56
IMAGE:180851	180851	GD	45	5,36E-03	−2,82	0,61
MGC5566	53119	GD	43	1,74E-03	3,18	1,85
D21S2056E	740140	GD	36	2,44E-03	3,08	1,72
IMAGE:729489	729489	GD	31	1,28E-03	3,28	2,95
KIAA1737	122063	GD	29	1,78E-03	3,18	1,67
MLH3	250771	GD	21	8,59E-03	2,66	1,59
IMAGE:53081	53081	GD	18	3,35E-03	2,98	1,77
TERF1	135773	GD	16	2,81E-02	2,22	1,45
DCXR	724596	GD	15	3,39E-03	−2,97	0,61
IMAGE:136686	136686	GD	15	6,29E-02	1,87	1,48
FLJ11127	668625	MNG	88	2,30E-06	4,90	1,54
RODH	471641	MNG	83	1,51E-05	4,46	2,57
MGC17299	713422	MNG	59	6,54E-05	4,10	1,37
NR2F2	72744	MNG	57	3,10E-04	3,69	1,40
ZNF83	486356	MNG	50	3,72E-04	3,64	1,32
IMAGE:744505	744505	MNG	43	2,51E-04	3,74	1,38
IMAGE:745187	745187	MNG	42	1,15E-03	−3,31	0,78
CAV1	309645	MNG	41	3,38E-04	3,66	1,59
CNTN6	257773	MNG	41	2,34E-04	3,76	1,38
C10orf116	740941	MNG	40	2,82E-04	3,71	1,72
CALM1	594510	MNG	39	1,21E-03	−3,30	0,77
IMAGE:726782	726782	MNG	39	3,97E-04	3,62	1,38
IMAGE:250463	250463	MNG	28	6,76E-03	2,74	1,29
IMAGE:731726	731726	MNG	28	3,90E-04	3,62	1,52
TTC17	665668	MNG	28	5,24E-04	3,54	1,31
PRKCA	768246	MNG	25	9,12E-04	−3,38	0,71
NR4A1	1073288	MNG	19	1,88E-03	−3,16	0,64
PDLIM1	135689	MNG	14	9,76E-04	3,36	1,48
IMAGE:731292	731292	MNG	11	2,65E-02	−2,24	0,68
SLC17A2	207920	MNG	9	2,70E-02	−2,23	0,67
MLL	80688	OTA	76	1,00E-07	−8,18	0,55
ALDH1A3	486189	OTA	67	1,00E-07	6,38	1,79
CLECSF12	258865	OTA	63	1,00E-07	7,66	3,51
IMAGE:485104	485104	OTA	60	1,00E-07	7,32	2,25
DCN	666410	OTA	49	1,00E-07	−7,19	0,50
PROZ	430471	OTA	47	1,00E-07	7,05	2,76
CHRDL2	485872	OTA	46	1,00E-07	6,72	1,58
B3GNTL1	53341	OTA	39	9,00E-07	5,10	2,04
SNRP70	729971	OTA	39	1,00E-07	−7,00	0,52
HSPC159	365045	OTA	38	1,00E-07	6,86	1,70
C4A	724366	OTA	32	1,00E-07	−6,30	0,39
SLC6A8	725877	OTA	31	1,00E-07	−7,00	0,23
OBSCN	730926	OTA	29	1,00E-07	6,95	1,55
IMAGE:731616	731616	OTA	27	1,00E-07	6,88	2,42
DC12	724895	OTA	24	1,00E-07	6,67	1,71
UBA52	530069	OTA	16	4,40E-06	−4,75	0,29
KCNQ2	179534	OTA	14	1,00E-07	6,43	1,77
HCNGP	365934	OTA	11	1,92E-05	4,40	1,40
C9orf72	726849	OTA	10	4,40E-06	−4,75	0,69
GNAI1	753215	OTA	6	8,90E-06	4,59	1,48
SDHA	40304	OTC	95	1,00E-07	6,29	3,16
DNALI1	782688	OTC	72	1,00E-07	−5,53	0,24
IMAGE:382423	382423	OTC	50	1,50E-06	5,00	3,52
ATP11C	667991	OTC	47	2,30E-05	4,36	2,17
C1QBP	173371	OTC	46	2,70E-06	4,86	2,47
DCN	666410	OTC	43	6,80E-06	−4,65	0,29
MT1F	78353	OTC	41	3,70E-06	−4,79	0,16
KIAA0543	175080	OTC	38	9,15E-05	4,01	2,03
FBXO46	471664	OTC	37	1,67E-05	4,44	2,85
CD33	1917430	OTC	35	1,06E-05	4,55	3,71
TH	813654	OTC	32	8,32E-04	3,41	1,95
IMAGE:742061	742061	OTC	31	1,99E-05	4,39	2,73
IDH3B	723755	OTC	27	1,90E-05	4,41	2,27
IMAGE:359454	359454	OTC	23	1,31E-05	−4,50	0,27
SST	39593	OTC	23	1,34E-03	3,26	1,81
DHRS6	364412	OTC	20	4,03E-04	−3,61	0,48
IMAGE:136933	136933	OTC	19	6,25E-03	2,77	2,19
TPM2	740620	OTC	18	1,59E-05	−4,45	0,30
TM7SF3	666928	OTC	14	3,58E-04	3,65	2,00
PREB	740347	OTC	5	1,29E-02	−2,51	0,33
LRRIQ2	487152	OTUM	59	1,29E-04	3,92	2,01
LRRC28	26519	OTUM	53	2,04E-04	−3,80	0,43
NDUFC1	796513	OTUM	53	2,65E-03	−3,05	0,61
NUMBL	1855110	OTUM	53	2,94E-04	−3,70	0,56
IMAGE:667527	667527	OTUM	48	2,31E-04	3,77	2,45
MAK	382002	OTUM	43	3,71E-04	3,64	2,14
PCGF4	740457	OTUM	43	3,06E-04	3,69	2,61
IFITM3	713623	OTUM	42	1,74E-02	2,40	1,63
CLGN	1049033	OTUM	40	1,52E-03	3,23	1,86
DEFB1	665086	OTUM	40	9,17E-04	3,38	2,79
IMAGE:669098	669098	OTUM	35	9,47E-04	3,37	1,85
GNAS	382791	OTUM	31	6,58E-04	3,47	2,75
PTTG1	742935	OTUM	29	7,53E-03	−2,71	0,52
USP14	376462	OTUM	28	9,21E-03	−2,64	0,65
SNF7DC2	730401	OTUM	25	1,36E-02	2,49	1,51
C21orf84	730814	OTUM	19	1,24E-03	3,29	2,08
TCTE3	136862	OTUM	19	2,48E-02	−2,27	0,69
TH	813654	OTUM	19	3,81E-02	−2,09	0,69
BAK1	235938	OTUM	16	1,12E-03	3,32	1,80
PAX6	230882	OTUM	12	1,28E-02	−2,52	0,66
CDH3	359051	PTC	99	1,00E-07	9,16	3,95
DPP4	343987	PTC	98	1,00E-07	8,72	4,22
CLDN1	594279	PTC	86	1,00E-07	7,88	8,98
SEP-02	729542	PTC	83	2,60E-01	1,13	1,20
ABCC3	208097	PTC	74	1,00E-07	6,30	3,48
ECM1	301122	PTC	67	1,00E-07	8,24	6,00
TSC	745490	PTC	60	1,00E-07	7,70	4,65
QPCT	711918	PTC	57	1,00E-07	7,27	3,36
ADAMTS9	376153	PTC	48	6,40E-06	4,66	1,95
IMAGE:687667	687667	PTC	48	1,00E-07	7,10	3,74
SLPI	378813	PTC	46	1,00E-07	7,03	2,98
MATN1	1624260	PTC	43	1,00E-07	−6,74	0,42
CITED1	265558	PTC	40	1,00E-07	6,84	4,88
CCND1	324079	PTC	38	1,00E-07	6,39	2,55
IMAGE:136976	136976	PTC	23	1,00E-07	6,15	2,11
PET112L	743125	PTC	20	1,00E-07	6,27	1,96
DAF	627107	PTC	16	1,00E-07	5,68	1,89
GSS	140405	PTC	7	1,00E-07	5,73	2,05
CASP3	823680	PTC	5	6,00E-07	5,20	2,08
CD9	727251	PTC	1	1,45E-05	4,47	1,57
IMAGE:687667	687667	TUM	94	1,00E-07	6,33	3,90
CREB3L2	136399	TUM	63	4,90E-06	−4,73	0,48
EIF1AY	380394	TUM	55	4,02E-05	−4,22	0,57
DJ462O23.2	738970	TUM	51	1,64E-05	4,44	1,63
CITED1	265558	TUM	49	1,21E-05	4,51	3,53
KLF1	208991	TUM	46	3,72E-05	−4,24	0,43
SNCB	50202	TUM	45	4,68E-05	4,18	1,99
FLJ10748	726830	TUM	44	4,24E-05	4,21	1,71
ANK2	2139315	TUM	43	1,31E-03	3,27	1,68
IGFBP3	269873	TUM	39	9,68E-05	4,00	2,06
WDR1	714196	TUM	37	2,06E-04	−3,80	0,65
IFNAR2	123950	TUM	36	2,56E-04	−3,74	0,61
SCNN1A	741305	TUM	36	2,37E-05	−4,35	0,41
TSC	745490	TUM	27	1,95E-04	3,81	2,61
NBL1	503874	TUM	24	2,47E-04	−3,75	0,35
ETF1	146976	TUM	18	6,36E-04	3,48	1,63
ICOSLG	2074228	TUM	18	1,12E-03	3,32	1,55
NCB5OR	743367	TUM	15	2,03E-03	−3,14	0,56
ZNF415	23939	TUM	14	4,94E-03	−2,85	0,63
KLK2	1102600	TUM	9	1,39E-03	−3,25	0,66

AT: autoimmune thyroiditis; FTA: macrofollicular thyroid adenoma; FTAb: microfollicular thyroid adenoma; FTC: follicular thyroid carcinoma; GD, Graves' disease; MNG: multinodular goiter; OTA: oncocytic thyroid adenoma; OTC: oncocytic thyroid carcinoma; PTC: papillary thyroid carcinoma; TUM: tumor of uncertain malignancy; and OTUM:, oncocytic tumor of uncertain malignancy.

### Class prediction from binary and from complete training

**Figure 2:**
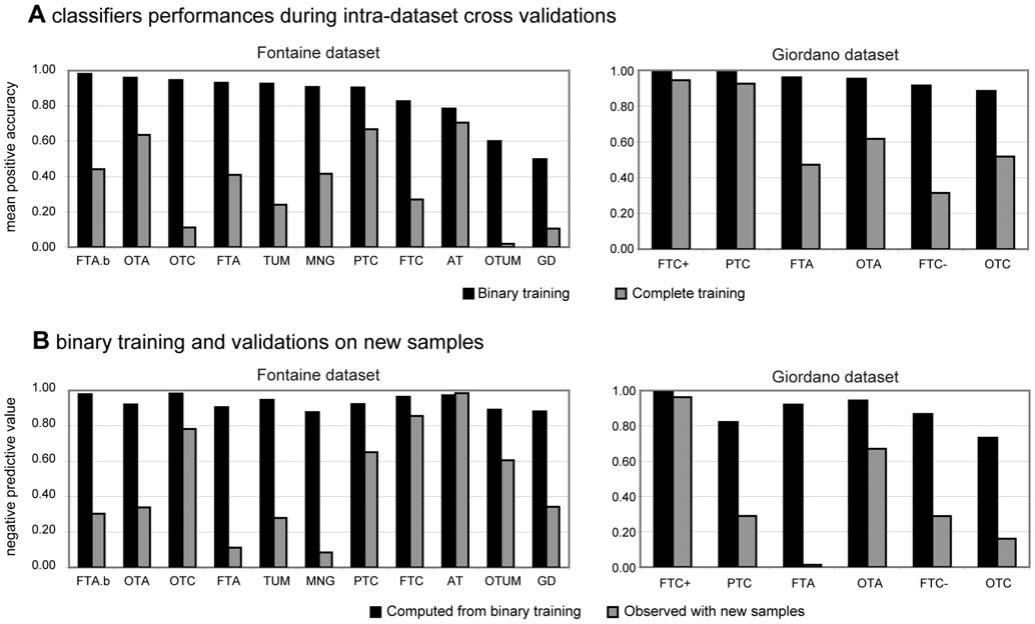
Class prediction of the samples. **Panel A** shows the mean positive accuracy of classifiers during intra-dataset cross-validations for the two main datasets, i.e. the Fontaine dataset and the Giordano dataset. Each classifier is composed of 20 genes selected by the Greedy Pairs algorithm and using Diagonal Linear Discriminant Analysis for training and prediction. Some classifiers were trained with samples from the selected class and the normal tissue, i.e. with binary training (black bars). Other classifiers were trained with all the samples of the dataset, i.e. with complete training (gray bars). **Panel B** shows the class prediction of new samples by binary-trained classifiers in the two datasets. All the new samples come from the same dataset and should have been predicted as negative. The negative predictive value which was deduced from binary training (black bars) is compared to observations on the new samples (gray bars). WT: wild type tissue; PTC: papillary thyroid carcinoma; FTA: macrofollicular thyroid adenoma; FTAb: microfollicular thyroid adenoma; MNG, multinodular goiter; FTC: follicular thyroid carcinoma (FTC+: with Pax8/PPARγ translocation; FTC-: without Pax8/PPARγ translocation); OTA: oncocytic thyroid adenoma; OTC: oncocytic thyroid carcinoma; TUM: tumor of uncertain malignancy; OTUM: oncocytic tumor of uncertain malignancy; GD, Graves' disease; and AT, autoimmune thyroïditis.

We compared classifiers defined from many classes, i.e. the entire dataset (complete training), and two classes, i.e. one pathological class *versus* normal thyroid tissue (binary training). The predictive accuracy for the samples of a class, i.e. the positive accuracy, was always higher after binary training than after complete training in the two main datasets (Figure 2A). With regard to complete training, only two classes in each dataset had similar performances: PTC and AT in the Fontaine dataset, FTC+ and PTC in the Giordano dataset.

The classifiers defined by complete training in the Fontaine dataset were evaluated in five other thyroid datasets (Table 4). With respect to the positive accuracy computed from intra-dataset cross-validations, the PTC, FTC, FTA and OTC classifiers turned out to be more accurate than expected. The OTA classifier was less accurate than expected, with a value of 0.59 *versus* 0.64, but only one other dataset contained this class of thyroid tissue.

**Table 4:** Inter-dataset cross-validations. Positive accuracies of the classifiers defined by the Fontaine dataset in five other datasets.

DATASET	PTC	FTC	FTA	OTC	OTA
Giordano *et al*. 2006	0.93	0.40	0.48	0.64	0.59
Weber *et al*. 2005	-	0.77	0.96	0.72	-
Jarzab *et al*. 2005	1.00	-	-	-	-
Reyes *et al*. 2006	1.00	-	-	-	-
He *et al*. 2005	1.00	-	-	-	-

FTA: macrofollicular thyroid adenoma; FTC: follicular thyroid carcinoma; OTA: oncocytic thyroid adenoma; OTC: oncocytic thyroid carcinoma; and PTC: papillary thyroid carcinoma.

### Functional analysis of cross-validated differential genes

**Figure 3:**
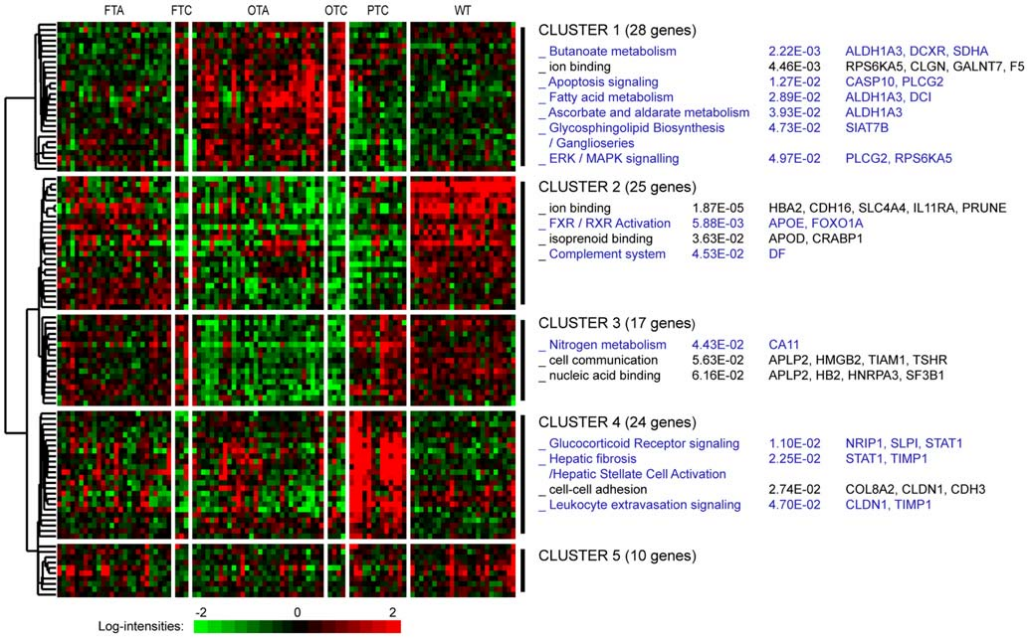
Hierarchical clustering of cross-validated differential genes. The heatmap shows gene-expression levels in the Fontaine dataset for the tissue classes common to the Giordano dataset (104 genes). Functional enrichments are shown for five identified gene clusters on the right, followed by p-values and the genes involved. The black text represents Level 3 Gene Ontology terms; the blue text represents canonical Ingenuity pathways. Gene profiles of the differential genes reflect the class similarities observed. WT: wild type tissue; PTC: papillary thyroid carcinoma; FTA: macrofollicular thyroid adenoma; FTC: follicular thyroid carcinoma; OTA: oncocytic thyroid adenoma; and OTC: oncocytic thyroid carcinoma.

In the two largest datasets containing the main kinds of thyroid lesions [5], [15], we searched for cross-validated differential genes. The two lists of differential genes had an intersection of 104 genes that separated five gene clusters from the hierarchical clustering of classes shared by the two datasets (Figure 3 and Table 5). The functions and pathways identified were mainly related to oncocytic and papillary tumors (Clusters 1, 3 and 4). Gene ontologies were associated with oxido-reductive mechanisms (Cluster 1, 28 genes), nitrogen metabolism and cell communication (Cluster 3, 17 genes), glucocorticoid receptor signaling and leukocyte extravasation (Cluster 4, 24 genes). A cluster of genes showed a specific profile for tumors of malignant potential, for which the transcriptional factors involved in the primary anabolic and catabolic cell pathways were down-regulated (Cluster 2, 25 genes). However in Cluster 2, the heterogeneous profiles of some classes (FTA) were suspected of containing molecular subgroups whereas the profiles for WT were almost homogeneous. A cluster of 10 genes (Cluster 5) was not clearly specific to the tissue classes as their profiles differed between the two datasets. In the Fontaine dataset, 52% of the cross-validated genes belonged to at least two of the 11 classes of lesion (data not shown).

**Table 5:** Cross validated differential genes from the two main datasets (n = 104).

Cluster	Symbol	UGCluster	Clone ID	P-value	P-value
				*(Fontaine et al. 2008)*	*(Giordano et al. 2006)*
1	CASP10	Hs.5353	241481	1,00E-07	1,00E-07
1	CLGN	Hs.86368	1049033	1,00E-07	1,00E-07
1	DEFB1	Hs.32949	665086	1,00E-07	1,00^E^-07
1	NTRK2	Hs.494312	2048801	1,00E-07	1,00^E^-07
1	SDHA	Hs.440475	40304	1,00E-07	1,00^E^-07
1	HSPC159	Hs.372208	365045	1,00E-07	3,00^E^-07
1	ALDH1A3	Hs.459538	486189	1,00E-07	8,00^E^-07
1	PLCG2	Hs.413111	201467	1,00E-07	9,00^E^-07
1	ARVCF	Hs.370408	40633	1,00E-07	1,50^E^-06
1	ELK4	Hs.497520	236155	1,00E-07	1,50^E^-06
1	DCI	Hs.403436	667892	1,00E-07	1,70^E^-06
1	DIO1	Hs.251415	296702	1,00E-07	3,50^E^-06
1	CKS2	Hs.83758	725454	2,00E-07	9,00^E^-07
1	ID1	Hs.504609	1087348	2,00E-07	3,90^E^-06
1	MRPS11	Hs.111286	471574	4,00E-07	1,00^E^-07
1	ST6GALNAC2	Hs.592105	823590	6,00E-07	8,00^E^-07
1	GFPT2	Hs.696497	485085	9,00E-07	1,00^E^-07
1	PITPNC1	Hs.591185	364436	9,00E-07	2,00^E^-07
1	HRK	Hs.87247	767779	1,00E-06	1,00^E^-07
1	MAPRE2	Hs.532824	383868	1,20E-06	1,00^E^-07
1	DOK5	Hs.656582	25664	1,30E-06	1,00^E^-07
1	RPS6KA5	Hs.510225	258966	1,40E-06	1,00^E^-07
1	DCXR	Hs.9857	724596	3,00E-06	1,00^E^-07
1	ODAM	Hs.143811	364706	3,30E-06	1,00^E^-07
1	KLK2	Hs.515560	1102600	5,00E-06	1,30^E^-06
1	F5	Hs.30054	433155	6,60E-06	1,00^E^-07
1	MFAP3L	Hs.593942	726821	7,60E-06	1,00^E^-07
1	GALNT7	Hs.548088	381854	1,10E-05	1,00^E^-07
2	AHNAK	Hs.502756	238840	1,00E-07	1,00^E^-07
2	APOD	Hs.522555	838611	1,00E-07	1,00^E^-07
2	CAMK2N1	Hs.197922	173820	1,00E-07	1,00^E^-07
2	CFD	Hs.155597	666128	1,00E-07	1,00^E^-07
2	FCGBP	Hs.111732	154172	1,00E-07	1,00^E^-07
2	FOXO1	Hs.370666	151247	1,00E-07	1,00^E^-07
2	GLT8D2	Hs.631650	365271	1,00E-07	1,00^E^-07
2	MATN2	Hs.189445	28584	1,00E-07	1,00^E^-07
2	PRUNE	Hs.78524	364324	1,00E-07	1,00^E^-07
2	SLC4A4	Hs.5462	787938	1,00E-07	1,00^E^-07
2	PAX8	Hs.469728	545475	1,00E-07	3,00^E^-07
2	HSD17B6	Hs.524513	471641	1,00E-07	1,30^E^-06
2	HBB	Hs.523443	469549	1,00E-07	2,60^E^-06
2	APOE	Hs.654439	1870594	1,00E-07	3,70^E^-06
2	CRABP1	Hs.346950	739193	1,00E-07	4,20^E^-06
2	ZNF76	Hs.388024	745003	1,00E-07	4,70^E^-06
2	C20orf19	Hs.187635	366032	1,00E-07	5,00^E^-06
2	DEPDC6	Hs.112981	669318	2,00E-07	1,00^E^-07
2	CDH16	Hs.513660	726763	5,00E-07	1,00^E^-07
2	HBA2	Hs.654744	469647	6,00E-07	1,80^E^-06
2	SASH1	Hs.193133	31120	1,40E-06	1,00^E^-07
2	HEXIM1	Hs.15299	342551	2,20E-06	1,00^E^-07
2	GJA1	Hs.74471	839101	2,30E-06	1,40^E^-06
2	TIMP3	Hs.644633	501476	4,30E-06	4,80^E^-06
2	IL11RA	Hs.591088	1101773	5,50E-06	7,00^E^-07
3	APLP2	Hs.695920	549054	1,00E-07	1,00^E^-07
3	DCN	Hs.694789	666410	1,00E-07	1,00^E^-07
3	FRMD4B	Hs.371681	669564	1,00E-07	1,00^E^-07
3	HMGB2	Hs.434953	884365	1,00E-07	1,00^E^-07
3	IGF2BP2	Hs.35354	743774	1,00E-07	1,00^E^-07
3	LOC400451	Hs.27373	667174	1,00E-07	1,00^E^-07
3	NPAL3	Hs.523442	738970	1,00E-07	1,00^E^-07
3	PBXIP1	Hs.505806	366042	1,00E-07	1,00^E^-07
3	PDGFRL	Hs.458573	139242	1,00E-07	1,00^E^-07
3	PDLIM1	Hs.368525	135689	1,00E-07	1,00^E^-07
3	PSAT1	Hs.494261	366388	1,00E-07	1,00^E^-07
3	SF3B1	Hs.632554	739247	1,00E-07	1,00^E^-07
3	TIAM1	Hs.517228	23612	1,00E-07	1,00^E^-07
3	TSHR	Hs.160411	565317	1,00E-07	1,00^E^-07
3	FAM111A	Hs.150651	137454	1,00E-07	4,00^E^-07
3	CA11	Hs.428446	282587	3,00E-07	1,00^E^-07
3	HNRPA3	Hs.516539	365349	5,00E-07	2,60^E^-06
4	ABCC3	Hs.463421	208097	1,00E-07	1,00^E^-07
4	CDH3	Hs.461074	359051	1,00E-07	1,00^E^-07
4	CITED1	Hs.40403	265558	1,00E-07	1,00^E^-07
4	CLDN1	Hs.439060	594279	1,00E-07	1,00^E^-07
4	DPP4	Hs.368912	343987	1,00E-07	1,00^E^-07
4	DUSP5	Hs.2128	33285	1,00E-07	1,00^E^-07
4	NOTCH2	Hs.487360	1641901	1,00E-07	1,00^E^-07
4	QPCT	Hs.79033	711918	1,00E-07	1,00^E^-07
4	S100A4	Hs.654444	868577	1,00E-07	1,00^E^-07
4	SCEL	Hs.534699	668239	1,00E-07	1,00^E^-07
4	SLPI	Hs.517070	378813	1,00E-07	1,00^E^-07
4	TESC	Hs.525709	745490	1,00E-07	1,00^E^-07
4	TIMP1	Hs.522632	162246	1,00E-07	1,00^E^-07
4	MGAT3	Hs.276808	731060	1,00E-07	8,00^E^-07
4	MDK	Hs.82045	309009	2,00E-07	1,00^E^-07
4	BID	Hs.591054	128065	6,00E-07	1,00^E^-07
4	NRIP1	Hs.155017	38775	6,00E-07	1,00^E^-07
4	COL8A2	Hs.353001	486204	1,00E-06	1,00^E^-07
4	MRC2	Hs.7835	235882	1,60E-06	9,00^E^-07
4	STAT1	Hs.699271	110101	2,10E-06	1,00^E^-07
4	ARMCX3	Hs.592225	251452	2,40E-06	1,00^E^-07
4	LAMB3	Hs.497636	1103402	9,90E-06	1,00^E^-07
4	CD55	Hs.527653	627107	1,30E-05	1,00^E^-07
5	DUS4L	Hs.97627	726904	1,00E-07	1,00^E^-07
5	ESRRG	Hs.444225	44064	1,00E-07	1,00^E^-07
5	ISG20	Hs.459265	740604	1,00E-07	1,00^E^-07
5	ROR2	Hs.644776	1089025	1,00E-07	1,00^E^-07
5	S100A11	Hs.417004	143957	1,00E-07	1,00^E^-07
5	SEMA4D	Hs.655281	210587	1,00E-07	1,00^E^-07
5	CD58	Hs.34341	2103105	1,00E-07	2,00^E^-07
5	IRF9	Hs.1706	724588	1,10E-06	3,20^E^-06
5	MCOLN1	Hs.631858	726156	2,00E-06	2,00^E^-07
5	ITGB7	Hs.654470	1337232	1,09E-05	2,90^E^-06

### Independent validation of selected genes by real-time RT-PCR

**Figure 4:**
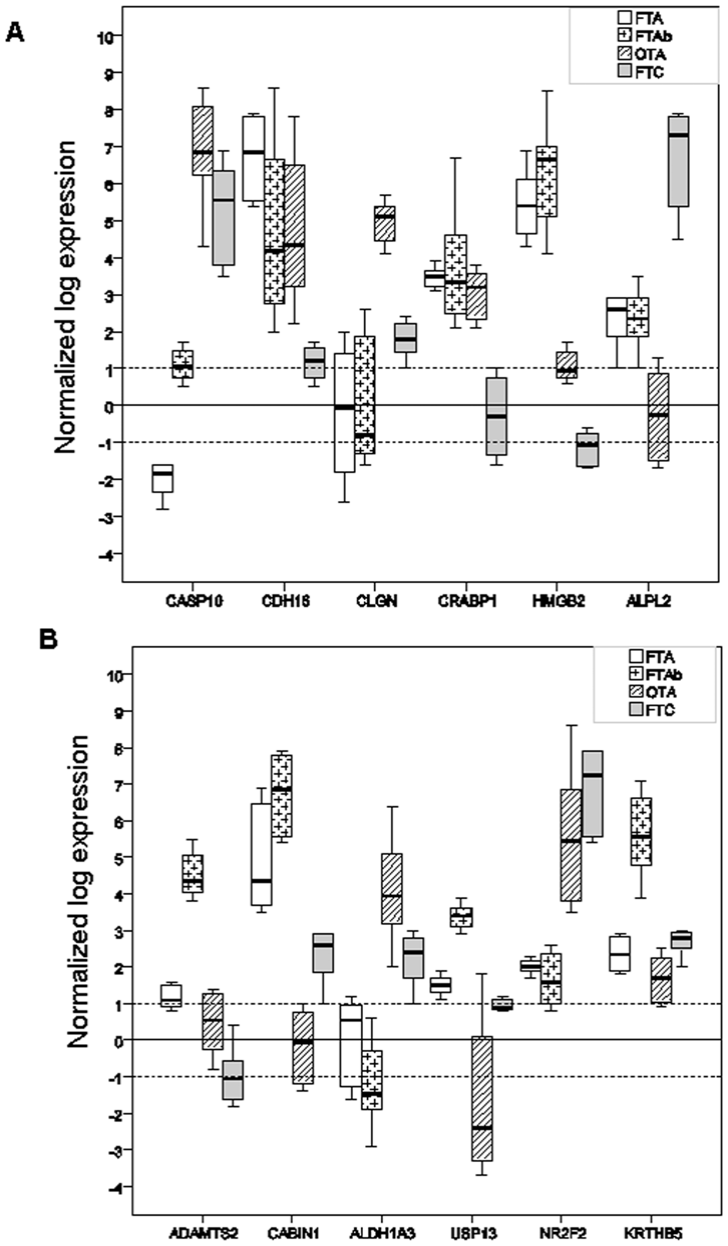
Gene expression of selected markers. Differential expression of 12 genes measured by real-time quantitative RT-PCR on 32 new follicular thyroid tumors (8 FTA, 8 FTAb, 8 OTA and 8 FTC). The upper and the lower limits of each box represent the upper and the lower quartiles, respectively. The bold lines represent medians. The expression of ADAMTS2, CABIN1, ADLH1A3, USP13, NR2F2, KRTHB5, CASP10, CDH16, CLGN, CRABP1, HMGB2 and ALPL2 genes was referred to the β-globin expression level. Significances of differential expression between the classes were assessed by t-tests and those over the classes by F-tests. FTA: macrofollicular thyroid adenoma; FTAb: microfollicular thyroid adenoma; FTC: follicular thyroid carcinoma; and OTA: oncocytic thyroid adenoma.

To test the relevance of some markers selected by cross-validation between the two main datasets, we measured the expression levels of six differential genes, CASP10, CDH16, CLGN, CRABP1, HMGB2 and ALPL2, on 32 new follicular tumor samples, including eight samples each of FTA, FTAb, OTA and FTC (Figure 4A). For all six genes, the expression levels differentiated between follicular adenomas (macro- and micro-follicular) and follicular carcinomas (t-test, p<0.05). In all cases except one (CASP10, F-test, p<0.05), the gene expression level differentiated not only between macro- and micro-follicular adenomas but also between follicular adenomas and oncocytic adenomas, as in the case of CDH16 and CRABP1 genes.

To test the relevance of our microarray analysis in identifying subclasses of adenomas, we measured the expression levels of six differential genes from the microfollicular adenoma subclass (ADAMTS2, CABIN1, ALDH13, USP13, NR2F2 and KRTHB5), on the same 32 follicular thyroid samples mentioned above (Figure 4B). Except for NR2F2 (p<0.954), all genes were differentially expressed not only between the three subclasses of adenomas (t-test, p<0.05), but also between the four groups of follicular tumors (F-tests, p<0.05 for ADAMTS2, CABIN1, ALDH13, USP13; p = 0.06 for KRTHB5).

### Mutational status of follicular tumors

The mutational status of three genes (TSHR, GNAS and NRAS) was investigated on 59 solitary follicular adenomas (43 from the microarray study, and 16 from the RT-PCR study) to compare these to the expression profile of FTA samples and evaluate their functional status. To identify the role of the PAX8-PPARG translocation in the expression profile of the FTC class, the rearrangement status for PAX8-PPARG was explored on 11 FTC samples (three samples from the microarray study, and eight samples from the RT-PCR study).

In the follicular adenoma samples, we identified mutations in the TSHR gene in seven of the 59 samples (12%): six of which were macrofollicular adenomas from the microarray study and one was a macrofollicular adenoma from the RT-PCR study. All the mutations were situated in the seventh transmembrane domain of the TSH receptor (from codon 619 to codon 633). Our results are in accordance with the mutational status of the TSH receptor gene described in the literature, especially concerning the macrofollicular subtype [19]. One macrofollicular adenoma presented a mutation in codon 201 of the GNAS gene. None of the microfollicular adenomas showed TSHR and GNAS mutations in the four exons explored. Five microfollicular adenomas (four from the microarray study, and one from the RT-PCR study) were positive for the NRAS mutation in codon 61.

No PAX8-PPARG translocation was found in any of the FTC samples studied.

### Protein expression of selected differential genes in independent samples

**Figure 5:**
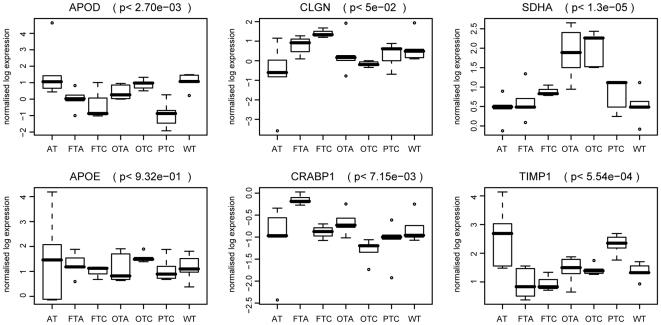
Protein expression of selected genes. The protein expression of six selected differential genes was measured on 49 new tissue samples. These independent samples represent six tissue classes that are common to the two main datasets (WT, PTC, FTA, FTC, OTA, and OTC). The AT class was added to compare the expression levels with a non-tumoral lesion. Seven samples were used for each class. Protein expression profiles of APOD, CLGN, SDHA, APOE, CRABP1 and TIMP1 are shown by box-plots for the different classes of tissue. Significances of differential expression over the classes were assessed by F-tests. P-values are shown between brackets. WT: wild type tissue; PTC: papillary thyroid carcinoma; FTA: macrofollicular thyroid adenoma; FTC: follicular thyroid carcinoma; OTA: oncocytic thyroid adenoma; and OTC: oncocytic thyroid carcinoma.

We collected 49 new tissue samples to measure the protein expression of six selected differential genes in the classes common to the two main datasets (FTA, FTC, OTA, OTC, PTC and WT), and also for the AT class as a non-tumoral control (Figure 5). There was significant differential expression between the classes (F-tests, p<0.05) for five proteins (SDHA, CLGN, APOD, CRABP1 and TIMP1). The APOE protein was not differentially expressed (p<0.932). Among the differentially expressed proteins, three had profiles similar to their gene profiles (SDHA, APOD and TIMP1), whereas two proteins (CLGN and CRABP1) showed some differences, as reported in previous studies of selected classes [20], [21]. The expression of the CLGN protein was higher in the FTA and FTC samples, but not in the oncocytic samples, contrary to the gene profile observed. The CRABP1 protein was differentially expressed between the FTA samples compared to the PTC and FTC samples, with a decreased expression of both RNA and protein in the malignant tumors. Considering the appurtenance of this gene to Cluster 2 (Figure 3), the expression of CRABP1 protein could serve to distinguish between the benign and malignant groups of thyroid tissue.

## Discussion

The clinical diagnosis of thyroid tumors is subject to significant inter- and intra-observer variations [22], [23]. Since 2001, various molecular markers for thyroid tumors have been proposed on the basis of microarray analysis. However, none of these markers has proved satisfactory in clinical practice, possibly because only a small number of thyroid tumor classes were initially taken into account. In our study, we analyzed six publicly available microarray datasets containing 347 thyroid tissue samples and defining as many as 12 classes of thyroid tumors. One of these datasets, the Fontaine dataset generated by our laboratory, comprised about half the total number of samples, including all the differentiated follicular thyroid lesions.

With regard to the 12 classes of thyroid tumor defined, we found that more than half of the 104 relevant and cross-validated differential genes were also related to other classes, such as AT, GD, TUM, FTAb, which were not shared by the two main datasets, i.e. the Giordano dataset and the Fontaine dataset. This may have affected the relevance of the signature in the classification of the follicular pathologies, explaining the low percentage of genes common to the two classifiers. Although the genes were selected from different sets of classes, discrepancies of some gene profiles, for example in Cluster 5, may be related to incomplete microarray platform compatibility or to differential cellular environment. Interestingly, most of the genes in Cluster 5 were involved in the process of vascular endothelial cell expansion[24]–[26], which may represent the stromal reaction. Despite the small number of FTC and OTC samples and the microarray platform being restricted to 9,216 probes, our classifier was able to validate the classification of PTC, FTC, FTA and OTC on five other datasets (Table 3). This demonstrated the relevance of our approach to the molecular classification of the main types of differentiated follicular thyroid pathology.

Unsupervised analysis of the class signatures showed a separation into three groups corresponding to benign, malignant, and oncocytic thyroid tumors (Figure 1). The pairwise distinctions of benign/normal and malignant tumors were consistent with published reports, as in the case of PTC and FTA [14]. The surprising proximity of the FTC and FTA classes in the Giordano dataset has already been discussed [4]. This could be related to the high homogeneity in the expression profiles of the FTC samples, which were positive for the PAX8-PPARG rearrangement and significantly related to the malignant profile of the PTC samples. Thus, the clustering of the FTC- and FTA classes appears to be related more to their heterogeneous expression profiles rather than to their tendency to tumoral behavior. These results strongly suggest the existence of molecular subclasses for the FTA and FTC- samples. Interestingly, the FTC samples in the Fontaine dataset, included in the malignant group, were negative for the PAX8-PPARG translocation.

Although the histologic diagnosis of oncocytic follicular tumors presents few problems, the two latest WHO classifications have debated whether these tumors should be considered as an independent class of tumors or as a variant of follicular tumors. This question is still relevant in view of the recent results of miRNA profiling that clearly revealed the segregation of oncocytic tumors from other conventional follicular tumors [27]. According to their specific gene-expression profiles as well as their mutational status for RAS and PAX8-PPARγ genes [7], oncocytic tumors should be considered as distinct from the other follicular tumors. The role of the “oncocytic group” signature in the appropriate classification of benign and malignant tumors is debatable. Considering the mitochondrial richness of follicular thyroid tumors, this signature may highlight the metabolic changes during tumorigenesis. Little is known about the etiology of oncocytomas but their signature is composed of numerous mitochondrial and metabolic genes, and the role played by these genes in tumor aggressivity has not yet been established [7], [20]. Interestingly, we found that microfollicular adenomas (FTAb) were related by their molecular profiles to oncocytic adenomas as well as to carcinomas. Relationships between thyroid metabolism and microfollicular lesions have been explored [28], but never in the context of mitochondrial functions. However, in a recent study, the galectin-3 profile associated with FTAb was reported to be related to its role in the regulation of mitochondrial stability [29]. Galectin-3 has been also involved in thyroid tumorigenesis through its anti-apoptotic activity [30]. Thus, the malignant potential of FTAb is still under discussion [31].

Evaluation of the performance of the classifiers used showed that poor performance was associated with the FTA subclasses in the two main datasets. In our study, the functional status of the FTA samples may have influenced the performance of the classifiers in discriminating true FTA from hyperfunctioning nodules. Only 12% of macrofollicular adenomas presented mutations in the TSH receptor or GNAS genes. These results correlated on microarray analysis with the expression profile of several genes previously associated with the hyperfunctional status of nodules [32]. Even though our FTA group represents a mix of adenomas in terms of functional status, the signature of follicular adenoma defined by our classifier are involved in other processes than iodine uptake and thyroid hormone secretion. As the selected genes are mainly associated to cell metabolism (CABIN1, CAMK2A, DAP, PDK4), signal transduction (ASGR1, PLXNB2, TBC1, PLCG1) and cell adhesion (COL18A1), we believe that the FTA signature we propose can discriminate true FTA from other follicular thyroid tumors. The relevance of constructing subclasses of follicular adenomas was tested on six cross-validated markers and six specific markers from our own analysis (Figure 4). Five specific markers enabled us to segregate micro- and macro-follicular adenomas from oncocytic adenomas and minimally invasive follicular carcinomas, while only one of the six cross-validated markers showed the same performance. Distinguishing between subclasses of adenoma allowed the identification of relevant molecular markers of follicular adenomas. These markers had never been differentially selected during previous microarray analyses of thyroid tumors. However, three of the markers (ADAMTS2, ALDH1A3 and CABIN1) had been proposed as prognostic markers for the recurrence of epithelial tumors [33]–[35].

The observation of six protein profiles highlights the difficulty of proposing immunomarkers for clinical practice. However, some markers may be relevant for the classification of differentiated follicular pathologies (FTA, FTC, PTC, OTA and OTC). It has been suggested that APOD, which has a specific protein profile for FTC and PTC, may modify the proliferative activity of cancer cells [36]. The differential expression of the folding protein calmegin (CLGN) with FTA and FTC samples may be associated with a difference in the abundance of the protein, as recently described in thyroid tumors [37]. Furthermore, increasing the number of thyroid tumor classes has revealed new aspects of some of the markers. Thus, TIMP1 and CRABP1 were not selected by our classifier but figured in the intersecting differential gene lists. Our data clearly questioned the relevance of TIMP1 as a marker of thyroid cancer. Since high protein levels were observed in the AT and PTC classes, TIMP1 expression may be more closely related to the frequently described lymphocytic infiltration in PTC [38]. Quantitative RT-PCR analysis on new follicular samples confirmed the upregulation of CRABP1 expression in all the adenomas as compared to follicular carcinomas. Underexpression of CRABP1 mRNA was relevant in distinguishing the FTC as well as PTC classes, as reported previously [39], [40]. However, our results differed at the protein level compared to a study on cold thyroid nodules where both the expression of CRABP1 mRNA and the protein were downregulated compared to normal surrounding tissue [41]. As our FTA samples were mostly non-functioning nodules, we postulate that this difference in protein level may be due to the histological class studied, macrofollicular adenomas in the Fontaine dataset, as opposed to the mix of follicular adenomas and adenomatous nodules in the other study. While all these markers can be used to complement current markers [42], the exploration of a large panel of protein markers to improve the differential diagnosis may rapidly become more cumbersome and less relevant than the study of the expression profile of selected genes, especially in the case of suspicious nodular thyroid lesions.

In conclusion, diagnostic tools defined on the basis of thyroid microarray data are more relevant when many samples and tissue classes are used, especially with the inclusion of oncocytic tumors. Our study has revealed additional potentially useful gene and protein markers allowing the classification of several thyroid pathologies. However, to identify early events of transformation, these markers should be viewed in the context of the biological processes in which they participate. We therefore believe that dedicated gene-expression profiling offers the most powerful method of identifying gene groups that correlate to nodular growth, thyroid dedifferentiation or malignancy, taking into account the potential contamination by other cell types such as activated lymphocytes. In this context, the molecular classification of microfollicular adenomas on fine needle aspiration biopsies should have a direct impact in the management of such tumors.

## Materials and Methods

### Samples and microarray datasets

We analyzed gene-expression data from six microarray datasets containing up to 12 classes of differentiated thyroid pathologies and normal thyroid tissue. The FTA samples were divided into two subclasses according to their follicular architecture, FTA (macrofollicular) and FTAb (microfollicular). The dominant nodule within the multinodular goiter (MNG) was investigated to explore the incidence of genetic or environmental events on the molecular signature of the class of follicular adenoma. When possible, the FTC class was also divided into two subclasses, FTC+ and FTC-, defined respectively by the presence or absence of the PAX8/PPARγ translocation in the samples. Atypical (TUM) and oncocytic features (OT) were individualized on criteria specified elsewhere [15], [20]. We also explored non-tumoral lesions such as those in Graves' disease (GD) and autoimmune thyroiditis (AT) to identify the immunological environment of the tumoral samples. This study is based on the Fontaine dataset generated by our laboratory, which comprises 12 subclasses containing a total of 166 anonymized human thyroid tissues. The five other datasets, particularly the Giordano dataset with 7 subclasses, were used to cross-validate the results of the molecular classification, the classifiers, and the functional analysis of differential genes [5], [43]–[45]. One dataset (Reyes *et al*.) has not yet associated citation. Details of the six datasets, containing a total of 347 samples, are summarized in Table 1.

The Fontaine and the Giordano datasets are referred as the main datasets. All the datasets, generated by various microarray platforms (Table 1), are publicly available. The Fontaine dataset (GSE6339), the He dataset (GSE3467) and the Reyes dataset (GSE3678) are hosted by the Gene Expression Omnibus (GEO) database of the U.S. National Center for Biotechnology Information. The Weber dataset was downloaded from European Bioinformatics Institute ArrayExpress database (E-MEXP-97). The two other datasets were downloaded from the authors' websites: the Giordano dataset (http://dot.ped.med.umich.edu:2000/pub/PPARG/index.html) and the Jarzab dataset (http://www.genomika.pl/thyroidcancer/PTCCancerRes.html).

### Data analysis

For the Fontaine dataset generated in our laboratory, hierarchical clustering of the data was computed on log-transformed, median gene-centered and normalized data as described elsewhere [15]. Normalized data from the Giordano and Jarzab datasets were downloaded from the authors' websites. For the remaining datasets, raw data were downloaded from the GEO or Array Express databases and normalized with BRB Array Tools v3.7.0b1 developed by Dr. Richard Simon. Since the data originated from three different but one-color platforms, global normalization to the median was used to center the gene-expression values in each array. Genes showing minimal variation across the set of arrays were excluded from the analysis (log-intensity variation P-value>0.15). Hierarchical clustering was done on centered genes by using the centered correlation distance and an average linkage method. Robustness and discrepancy indices were computed from 10,000 data perturbations.

For the two largest datasets [5], [15], we identified genes that were differentially expressed among the classes by using a multivariate 10,000-permutation test, to provide 95% confidence that the number of false discoveries did not exceed 1. Although F-statistics were used for each gene, the multivariate permutation test is non-parametric and does not require the assumption of Gaussian distributions. Functional enrichments in gene clusters were defined at 5% risk for Level 3 Gene Ontology terms by Fatigo tools (adjusted P-values, www.fatigo.bioinfo.cipf.es [46]) as compared to the whole genome, and for canonical pathways by Ingenuity Pathway Analysis tools as compared to the Ingenuity Pathways Knowledge database (www.ingenuity.com).

Classifiers were defined for each pathological class and trained either *versus* the wild type class (binary training) or the entire dataset (complete training). Twenty genes were selected by the Greedy Pairs algorithm, and Diagonal Linear Discriminant Analysis (DLDA) was used for training and sample [47]. Intra- and inter-dataset cross-validations were done by the 0.632+ method and DLDA respectively [48]. Since the classical accuracy coefficient is biased in large datasets containing unbalanced class sizes, we preferred to use the positive accuracy coefficient defined as the geometric mean of the sensitivity (i.e. the proportion of true positives in the samples belonging to the targeted class in the entire datasets) and the positive predictive value (i.e. the proportion of true positives in the samples belonging to the targeted class) [49].

### Quantitative RT-PCR analysis

An independent set of 32 follicular tumors, comprising 8 minimally invasive FTC and 24 follicular adenoma (8 OTA, 8 FTA and 8 FTAb), were subjected to real-time quantitative RT-PCR to test the relevance of several selected markers. The anonymized samples were obtained from the Department of Pathology of the University Hospital of Angers, France.

Total RNA was isolated using the TRIzol reagent (Invitrogen, Paisley, UK). RNA integrity was determined using a Bio-Analyzer 2100 (Agilent Technologies, Waldbronn, Germany). Reverse transcription was performed on 1 µg of RNA with Advantage RT-for-PCR kit (Clontech Laboratory, Palo Alto, CA, USA) following the manufacturer's recommendations. Real-time quantification was performed in a 96-well plate using the IQ SYBR Green supermix and Chromo4 (Biorad, Hercules, CA, USA) with the recommended protocol. Twelve genes were explored for their expression levels: CASP10, CDH16, CLGN, CRABP1, HMGB2, ALPL2, ADAMTS2, CABIN1, ALDH1A3, USP13, NR2F2 and KRTHB5. Data were normalized to the β-globin expression level. The significance of differential expression between classes was assessed by t-tests and that over the classes by F-tests. The primer sequences used in this study are specified in Table 6.

**Table 6:** Primer sequences used for real-time quantitative RT-PCR.

Gene	Forward Sequence	Reverse Sequence
**ADAMTS2**	5′-GGG-AAG-GAG-CAC-GTA-CAG-AA-3′	5′-CGT-GAT-CGT-GGT-ATT-CAT-CG-3′
**ALDH1A3**	5′-TCT-CGA-CAA-AGC-CCT-GAA-GT-3′	5′-GTC-CGA-TGT-TTG-AGG-AAG-GA-3′
**APLP2**	5′-GCT-GTC-GTT-CCG-GTT-ATG-TT-3′	5′-GAG-GGT-CTC-TCA-CGT-GCT-TC-3′
**CABIN1**	5′-AGG-CCC-TGG-AGG-TGT-ACT-TT-3′	5′-CCA-GCT-GAA-GAG-TGG-GAG-TC-3′
**CASP10**	5′-TCT-CAG-GAT-CAC-TGG-GCT-CT-3′	5′-ATG-AAG-GCG-TTA-ACC-ACA-GG-3′
**CDH16**	5′-CAA-GTC-ATG-AGG-TGG-TGG-TG-3′	5′-GAT-GGT-CAG-CAG-GAA-AGA-GC-3′
**CLGN**	5′-CAA-TGG-ACC-TGG-AAG-AGG-AA-3′	5′-TGA-CTT-TAT-CGG-CCC-ATC-TC-3′
**CRABP1**	5′-CAG-GAC-GGG-GAT-CAG-TTC-TA-3′	5′-CGC-CAA-ACG-TCA-GGA-TAA-GT-3′
**HMGB2**	5′-AGA-GGC-TGA-GGA-TTG-CGT-TA-3′	5′-GGG-GTC-TCC-TTT-ACC-CAT-GT-3′
**KRTHB5**	5′-AGC-TCT-CAG-GGA-CAA-GAC-CA-3′	5′-AAC-AGG-TTA-GCC-CAG-AAG-CA-3′
**NR2F2**	5′-TGC-CTG-TGG-TCT-CTC-TGA-TG-3′	5′-ATA-TCC-CGG-ATG-AGG-GTT-TC-3′
**USP13**	5′-GAC-CTG-CGA-GAA-AAC-CTC-TG-3′	5′-CAG-GAG-TGA-TGG-TTC-CCA-GT-3′
**β-GLOBIN**	5′-CAA-CTT-CAT-CCA-CGT-TCA-CC-3′	5′-ACA-CAA-CTG-TGT-TCA-CTA-GC-3′

### Detection of mutations and PAX8-PPARG rearrangement

The mutational status for the TSHR, GNAS and NRAS genes was determined for all the FTA and FTAb explored in studies by microarray (26 FTA and 17 FTAb) and RT-PCR (8 FTA and 8 FTAb). DNA was isolated from frozen tissues during the guanidium isothiocyanate procedure (TRIzol Reagent, Invitrogen Life Technologies). Exon 2 of the NRAS gene was amplified using primer sequences described elsewhere[50]. Exons 9 and 10 of the TSH receptor gene, and exons 8 and 9 of the Gs alpha gene were amplified using primer sequences previously specified [51]. PCR were performed on 5 µl DNA with the HotGoldstar DNA polymerase (Eurogentec, Seraing, Belgium) according to the manufacturer's recommendations. Amplified fragments were purified and directly sequenced on a CEQ 8000 apparatus, using the CEQ DTCS Quick Start Kit (Beckman Coulter, Fullerton, CA, USA) following the manufacturer's instructions.

The presence of the PAX8-PPARG translocation was evaluated as described elsewhere [52], on the three FTC samples in the microarray study and the eight FTC samples in the RT-PCR study. Quantification, degradation and DNA contamination of RNA were assessed using an RNA 6000 Nano Assay kit (Agilent Technologies, Palo Alto, CA, USA). After reverse transcription, we looked for the translocation in 5 µl cDNA, using primer sequences previously specified [5] and the HotGoldstar DNA polymerase (Eurogentec, Seraing, Belgium) according to the manufacturer's recommendations.

### Dot blot analysis

Two micrograms of protein corresponding to the TRIzol fraction of 49 independent and anonymized thyroid tissues were spotted onto nitrocellulose membranes at room temperature, using a Dot Blot apparatus (Biorad, Hercules, CA, USA) following the manufacturer's recommendations. These tissues, provided by the Department of Pathology of the University Hospital of Angers, differed from those used for the microarray and RT-PCR studies. Six groups of thyroid pathologies were defined according to WHO cytological criteria: AT, FTA, FTC, PTC, OTA and OTC and compared to normal thyroid tissue (WT). Seven samples were tested from each group and the assays were performed in duplicate. Seven primary antibodies (all from Abcam, Cambridge, UK) were used at specific dilutions: 1/750 for TIMP1, 1/1000 for APOD, SDHA, CLGN, CRABP1 and APOE, and 1/5000 for α-tubulin as a control. Peroxidase anti-rabbit or anti-mouse secondary antibodies were revealed using the ECL Plus reagent kit (ECL, Amersham, Chalfont, UK). Spots were quantified on a GelDoc XRS apparatus using Quantity One software (Biorad) and expressed in terms of the control value. Significances of differential expression over the classes were assessed by F-tests.
